# Efficacy of High-Dose and Low-Dose Simvastatin on Vascular Oxidative Stress and Neurological Outcomes in Patient with Acute Ischemic Stroke: A Randomized, Double-Blind, Parallel, Controlled Trial

**DOI:** 10.1155/2018/7268924

**Published:** 2018-04-18

**Authors:** Nattaphol Uransilp, Pannawat Chaiyawatthanananthn, Sombat Muengtaweepongsa

**Affiliations:** ^1^Neurology, Faculty of Medicine, Thammasat University, Pathum Thani, Thailand; ^2^Department of Applied Thai Traditional Medicine, Faculty of Medicine, Thammasat University, Pathum Thani, Thailand; ^3^Department of Internal Medicine, Faculty of Medicine, Thammasat University, Pathum Thani, Thailand

## Abstract

**Backgrounds:**

Stroke is the leading cause of death and long-term disability. Oxidative stress is elevated during occurrence of acute ischemic stroke (AIS). Soluble LOX-1 (sLOX-1) and NO are used as biomarkers for vascular oxidative stress that can reflect stabilization of atherosclerotic plaque. Previous study showed that simvastatin can reduce oxidative stress and LOX-1 expression.

**Objectives:**

To evaluate neurological outcomes and serum sLOX-1 and NO levels in patients with AIS treatment with low dose 10 mg/day and high dose 40 mg/day of simvastatin.

**Methods:**

65 patients with AIS within 24 hours after onset were randomized to treatment with simvastatin 10 mg/day or 40 mg/day for 90 days. Personal data and past history of all patients were recorded at baseline. The blood chemistries were measured by standard laboratory techniques. Serum sLOX-1 and NO levels and neurological outcomes including NIHSS, mRS, and Barthel index were tested at baseline and Day 90 after simvastatin therapy.

**Results:**

Baseline characteristics were not significantly different in both groups except history of hypertension. Serum sLOX-1 and NO levels significantly reduce in both groups (sLOX-1 = 1.19 ± 0.47 and 0.98 ± 0.37 ng/ml; NO = 49.28 ± 7.21 and 46.59 ± 9.36 *μ*mol/l) in 10 mg/day and 40 mg/day simvastatin groups, respectively. Neurological outcomes including NIHSS, mRS, and Barthel index significantly improve in both groups. However, no difference in NO level and neurological outcomes was found at 90 days after treatment as compared between low dose 10 mg/day and high dose 40 mg/day of simvastatin.

**Conclusion:**

High-dose simvastatin might be helpful to reduce serum sLOX-1. But no difference in clinical outcomes was found between high- and low-dose simvastatin. Further more intensive clinical trial is needed to confirm the appropriate dosage of simvastatin in patients with acute ischemic stroke. This trial is registered with ClinicalTrials.gov ID: NCT03402204.

## 1. Introduction

Ischemic stroke is the main etiology of disability in senile population and remains the third most common cause of death in the world [1]. Stroke has been the common cause of mortality in Thailand for decades [2–4]. The prevalence of stroke is one percent in Thai people aged more than 30 years [5] or 1.88 percent in Thai people aged more than 45 years [6]. Oxidative stress is defined as a disturbance in the prooxidant-antioxidant balance in favor of the prooxidant, leading to potential damage [7]. Oxidative stress is elevated during occurrence of acute ischemic stroke (AIS) [8, 9].

Previous study found that oxidized-low density lipoprotein (ox-LDL) and oxidative stress induce production of lectin-like oxidized low density lipoprotein receptor-1 (LOX-1) and cleavage some extracellular parts of LOX-1 into blood circulation, and it is called soluble LOX-1 (sLOX-1) [10]. The sLOX-1 is used for biomarker in patients with myocardial infarction (MI), coronary artery diseases (CAD), metabolic syndrome, or others [11, 12]. It is known that oxidized-LDL can lead to plaque instability by increasing vascular oxidative stress and by upregulation of matrix metalloproteinases (MMPs).

During 24 hours after onset of ischemic stroke, nitric oxide (NO) is mainly produced by activation of both inducible nitric oxide synthase (iNOS) and neuronal nitric oxide synthase (nNOS). These two subtypes of NO are considered as neurotoxic agents and supposed to become lower at 3 months after onset. In contrast, NO created by endothelial nitric oxide synthase (eNOS) demonstrates neuroprotective effect [13]. However, eNOS produces small amount of NO at the ultraearly stage of ischemic stroke. Simvastatin shows gainful effect for ischemic stroke by upregulation of eNOS activity [14].

Simvastatin is a cholesterol-lowering medication which acts by inhibiting hydroxymethylglutaryl-coenzyme A (HMG-CoA) reductase, hence used for the primary and secondary prevention of ischemic stroke. Simvastatin can inhibit activation of extracellular regulated kinase (ERK) 1/2 and proliferation of rat vascular smooth muscle cells [15], attenuation of inflammation, oxidative stress and plaque stabilization, and plaque thickness in type 2 diabetes patients [16]. Simvastatin can reduce oxidative stress through inhibiting nicotinamide adenine dinucleotide phosphate (NADPH) oxidase and reducing angiotensin type 1 (AT1) receptor. Therefore, overall effect of simvastatin beyond lowering cholesterol includes improving endothelial function, modulating thrombogenesis, attenuating inflammatory, and oxidative stress damage, and facilitating angiogenesis [17].

This study aims to investigate outcomes of simvastatin 10 mg/day and 40 mg/day on vascular oxidative stress and neurological outcomes in patients with acute ischemic stroke. We expect that the results of our study might have clinical implications for ischemic stroke prevention in future.

## 2. Material and Methods

### 2.1. Study Population

We recruited patients with acute ischemic stroke received at Thammasat University Hospital between April 2014 and December 2015. Patients who met the following inclusion criteria were eligible: 18 to 85 years old; diagnosis of an acute ischemic stroke; and ability to start the study drug within 24 hours after symptom onset. Patients were excluded if they had any of the following: contraindication to simvastatin; prestroke mRS score more than 1; conscious level > 2 scores on question 2 of NIHSS; hematocrit less than 0.25; blood sugar (BS) less than 60 mg/dl or more than 200 ml/dl or between 200 and 300 mg/dl and treated with diabetes drug until the BS levels are less than 200 mg/dl; acute myocardial infarction (AMI) or coronary heart disease (CHD) within 3 weeks; patient who receives lower-lipid level drug, that is, ezetimibe, fenofibrate, gemfibrozil, and niacin, or statin drugs, that is, atorvastatin and pitavastatin, and increasing liver enzyme level or liver disease. The study was registered in ClinicalTrials.gov. The clinical study registration number is NCT03402204.

### 2.2. Study Design

Patients with acute ischemic stroke were divided into 2 groups (simvastatin 10 mg/day and 40 mg/day). Personal and past medical history were recorded after the patients signed informed consent. Blood samples were collected from patients for measuring biomarkers in serum related to vascular oxidative stress, that is, sLOX-1 and NO, and neurological examination was done, that is, NIHSS, mRS, and Barthel's index scale at Day 0 and Day 90.

### 2.3. Blood Chemical Analysis

Peripheral venous blood samples of all 65 patients with acute ischemic stroke were obtained not later than 24 hours after onset. The sample was centrifuged 3,000 rpm at 4°C for 15 minutes. Serum samples were frozen at −80°C until analysis. Serum blood sugar, cholesterol, triglycerides, high density lipoprotein cholesterol (HDL-c), and low density lipoprotein cholesterol (LDL-c) were measured by standard laboratory techniques of Thammasat University Hospital. Serum sLOX-1 and NO levels were determined using commercially available enzyme-linked immunosorbent assay (ELISA) kits (R&D systems, MN, USA).

### 2.4. Ethical Consideration

The clinical study protocol was submitted to Ethical Committee of Faculty of Medicine, Thammasat University (number 1) for approval before conducting experiments. The number of approved protocol is MTU-EC-4-019/58.

### 2.5. Data Analysis

SPSS version 16.0 for Windows (Chicago, IL, USA) was used for statistical analysis. All data were presented as mean ± standard deviation (SD). The possible statistical differences among groups were tested using Mann–Whitney *U* test or Chi square test. The possible statistical difference among persons at Day 0 and Day 90 was tested using Wilcoxon test. A probability value of less than 0.05 was considered to be statistically significant.

## 3. Results

### 3.1. Baseline Clinical Characteristics

34 patients were treated with simvastatin 10 mg/day and 31 patients were treated with simvastatin 40 mg/day during the study period. Baseline characteristics were shown in Table 1. There were no significant differences in age, systolic or diastolic blood pressure, blood sugar, lipid profile, NIHSS score, and treatment with IV rtPA between the two groups but there was significant difference in medical history of hypertension.

As Table 2 showed there is no significant difference in serum sLOX-1 and NO level at baseline in patients with AIS between 2 groups.

### 3.2. Association of Serum sLOX-1, NO Levels, and Neurological Outcomes after Simvastatin Therapy

**Figure 1:**
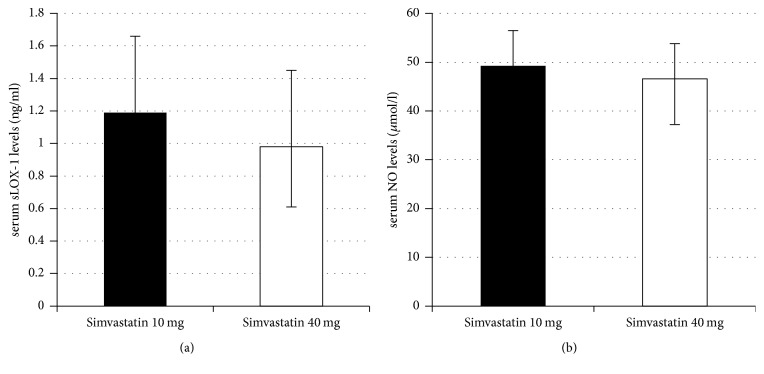
Serum sLOX-1 (a) and NO (b) levels of patients with acute ischemic stroke that compare simvastatin 10 mg/day and 40 mg/day at Day 90 after simvastatin treatment. ^*∗*^*P* < 0.05.

After 90 days of simvastatin treatment, serum sLOX-1 level was significantly reduced in simvastatin 40 mg/day group (*P* = 0.04) but there was no difference in NO level as compared between simvastatin 10 mg/day and simvastatin 40 mg/day group (Table 3 and Figure 1). When compared between Day 0 and Day 90 within each group (Table 4), both sLOX-1 and NO were significantly declined at Day 90 in both groups. NIHSS, mRS, and Barthel index were improved at Day 90 in both groups (Table 5, all *P* < 0.05). However, there was no difference in NIHSS, mRS, and Barthel index at Day 90 as compared between simvastatin 10 mg/day and simvastatin 40 mg/day group (Table 6).

## 4. Discussion

Acute ischemic stroke patients receiving simvastatin 10 and 40 mg/day for 90 days significantly decreased serum sLOX-1 and NO levels and improved neurological outcome. Simvastatin 40 mg/day group significantly reduced sLOX-1 level compared to simvastatin 10 mg/day at 90 days after treatment. According to the previous study of statin in patients with ischemic stroke, age did not affect any outcomes [18]. Patients aged between 18 and 85 years were included in our study.

The involvement of LOX-1 is a factor that affects development of atherosclerosis from several factors; for example, dyslipidemia played the major role in the upregulation of LOX-1 through ox-LDL stimulation [19], hyperglycemia increased LOX-1 upregulation in human endothelial cells via activation of reactive oxygen species (ROS) [20], and hypertension upregulated the expression of LOX-1 by induction of angiotensin II [21]. Previous studies have also found that sLOX-1 are significantly increased in obesity [22] and type 2 DM. The activation of LOX-1 affects atherosclerotic plaque formation and progression through dysfunction of endothelial cells [23], apoptosis of vascular smooth muscle cells [24], accumulation of lipids in macrophages [25], and production of matrix metalloproteinases [26]. Schwarz et al. reported that LOX-1 expression was induced 10-fold at ischemic core sites during experimental stroke [27]. Thus, activation of LOX-1 might facilitate the pathophysiological conditions leading to stroke.

The major findings of this study show that simvastatin significantly reduces serum sLOX-1 levels after 90 days of treatment. But only higher dose of simvastatin (40 mg/day) can decrease serum sLOX-1 at Day 90 of treatment. This finding reflects higher doses of simvastatin may be more useful in improving plaque stability and reduce risk for recurrent ischemic stroke than lower doses of simvastatin.

As mentioned above, serum NO demonstrates not only pros but also cons effects on patients with ischemic stroke. NO produced by iNOS and nNOS is among the cons while NO produced by eNOS is among the pros. From temporal ischemic stroke in our study, majority of NO is produced by iNOS and nNOS [13]. Decrement of NO at 90 days after onset should be a natural course of ischemic stroke. Simvastatin may upregulate eNOS leading to rising NO. However, eNOS usually produces a small amount of NO. Simvastatin may not be able to affect NO level in our study.

The mechanisms by which statins provide benefit to patients with acute ischemic stroke remain unclear and are likely multifactorial. Previous study indicates that statin has multiple effect beyond cholesterol lowering including improving endothelial function, modulating thrombogenesis, attenuating inflammatory and oxidative stress damage, and facilitating angiogenesis [17]. In animal model of stroke, statin shows benefits to reduction in infarct size [28] and improves neurological function and cerebral blood flow [29]. Recent study shows patients who take statin have 2.63 times greater probability of discharge home compared to untreated patients [30]. Our results are in agreement with previous studies that have shown improvement in functional outcome in stroke patients treated with statins.

There are several limitations to this study. First, this study was cross-sectional, thereby allowing the determination of associations but not formulation of risk predictions. In addition, the study populations were relatively small. Therefore, our findings need further investigation in prospective studies with larger sample size. Last, sLOX-1 and NO levels might be higher or lower in patients with ICAS than in general population. Therefore, a normal control group should be included in future studies to evaluate the degree of impact of the presence and severity of acute ischemic stroke. The low proportion of patients with neurological progression could be secondary to a selection bias because of the admission of patients with less severe symptoms. Last, neurological improvement in stroke patient could be from other factors than statin: age, NIHSS scale on admission, HbA1c level, and location of stroke [31].

## 5. Conclusion

Our study showed that high-dose simvastatin significantly reduced serum sLOX-1. However, no difference in clinical outcome between high-dose and low-dose simvastatin was found at 90 days after treatment. Further more intensive clinical trial is needed to confirm the appropriate dosage of simvastatin in patients with acute ischemic stroke.

## Figures and Tables

**Table 1:** Baseline characteristics of patients with acute ischemic stroke receiving simvastatin therapy.

Characteristic	Simvastatin 10 mg (*N* = 34)	Simvastatin 40 mg (*N* = 31)	*P* value
	Mean ± SD	Mean ± SD	
Age (years)	65.09 ± 13.93	62.4 ± 15.79	0.48
Female, *n* (%)	17 (50)	14 (45)	0.69
SBP (mmHg)	159.79 ± 28.78	147.19 ± 25.07	0.58
DBP (mmHg)	86 ± 16.92	83.84 ± 14.38	0.23
Blood Sugar (mg/dl)	109.8 ± 27.2	113.8 ± 21.70	0.51
Cholesterol (mg/dl)	182.15 ± 35.82	194.1 ± 44.49	0.24
Triglyceride (mg/dl)	101.38 ± 67.52	133.71 ± 103.69	0.14
HDL-c (mg/dl)	46.65 ± 10.68	49.16 ± 10.92	0.35
LDL-c (mg/dl)	120.38 ± 35.68	113.81 ± 21.70	0.73
NIHSS	6.47 ± 4.90	6.61 ± 3.79	0.89
rtPA therapy			
IV rtPA, *n* (%)	4 (11.76)	8 (25.8)	0.15
Medical history, *n* (%)			
Ischemic stroke	2 (5)	2 (6)	0.92
Atrial fibrillation or flutter	6 (17)	3 (9)	0.35
Valvular heart disease	1 (3)	0 (0)	0.34
Hypertension	23 (67)	12 (38)	0.02
Diabetes mellitus	3 (8)	5 (16)	0.37
Dyslipidemia	16 (47)	12 (38)	0.50
Others	1 (3)	3 (9)	0.90

*Note*. Systolic blood pressure (SBP), diastolic blood pressure (DBP), recombinant tissue plasminogen activator (rtPA), and intravenous route (IV).

**Table 2:** Serum sLOX-1 and NO levels in patients with acute ischemic stroke at baseline.

Parameter	Simvastatin 10 mg (*N* = 34) Mean ± SD	Simvastatin 40 mg (*N* = 31) Mean ± SD	*P* value
sLOX-1 levels (ng/ml)	1.42 ± 0.49	1.72 ± 0.55	0.21
NO levels (*µ*mol/l)	55.06 ± 9.26	51.72 ± 8.83	0.12

**Table 3:** Serum sLOX-1 and NO levels in patient with acute ischemic stroke received simvastatin therapy for 90 days.

Parameter	Simvastatin 10 mg (*N* = 34) Mean ± SD	Simvastatin 40 mg (*N* = 31) Mean ± SD	*P* value
sLOX-1 levels (ng/ml)	1.19 ± 0.47	0.98 ± 0.37	0.04
NO levels (*µ*mol/l)	49.28 ± 7.21	46.59 ± 9.36	0.2

**Table 4:** Compare serum sLOX-1 and NO levels in patient with acute ischemic stroke at 0 days and 90 days after simvastatin therapy.

Parameter	Simvastatin 10 mg (*N* = 34) Mean ± SD		*P* value	Simvastatin 40 mg (*N* = 31) Mean ± SD		*P* value
	Day 0	Day 90		Day 0	Day 90	
sLOX-1 levels (ng/ml)	1.42 ± 0.49	1.19 ± 0.47	<0.001	1.72 ± 0.55	0.98 ± 0.37	<0.001
NO levels (*µ*mol/l)	55.06 ± 9.26	49.28 ± 7.21	0.03	51.72 ± 8.83	46.59 ± 9.36	0.005

**Table 5:** Compare neurological outcome in patient with acute ischemic stroke 0 days and 90 days after simvastatin therapy.

Parameter	Simvastatin 10 mg (*N* = 34) Mean ± SD		*P* value	Simvastatin 40 mg (*N* = 31) Mean ± SD		*P* value
	Day 0	Day 90		Day 0	Day 90	
NIHSS	6.47 ± 4.90	3.15 ± 2.71	<0.001	6.61 ± 3.79	2.77 ± 3.04	<0.001
mRS	1.85 ± 1.16	1.26 ± 1.08	<0.001	1.65 ± 1.02	1.23 ± 1.18	0.002
Barthel Index	86.62 ± 12.54	90.74 ± 11.49	<0.001	87.90 ± 12.83	91.61 ± 13.25	0.004

*Note*. NIHSS, National Institutes of Health Stroke Scale; mRS, Modified Rankin Scale.

**Table 6:** Compare neurological outcome in patient with acute ischemic stroke at 90 days after simvastatin therapy.

Parameter	Simvastatin 10 mg (*N* = 34) Mean ± SD	Simvastatin 40 mg (*N* = 31) Mean ± SD	*P* value
NIHSS	3.15 ± 2.71	2.77 ± 3.04	0.60
mRS	1.26 ± 1.08	1.23 ± 0.99	0.88
Barthel Index	90.73 ± 11.49	91.61 ± 13.25	0.77

*Note*. NIHSS, National Institutes of Health Stroke Scale; mRS, modified Rankin Scale.

## Abbreviations

SBP: Systolic blood pressure
DBP: Diastolic blood pressure
rtPA: Recombinant tissue plasminogen activator
IV: Intravenous route
AIS: Acute ischemic stroke
LOX-1: Lectin-like oxidized low density lipoprotein receptor-1
sLOX-1: Soluble LOX-1
MI: Myocardial infarction
CAD: Coronary artery diseases
MMPs: Matrix metalloproteinases
iNOS: Inducible nitric oxide synthase
NO: Nitric oxide
HMG-CoA: Hydroxymethylglutaryl-coenzyme A
ERK: Extracellular regulated kinase
NADPH: Nicotinamide adenine dinucleotide phosphate
AT_1_: Angiotensin type 1
BS: Blood sugar
CHD: Coronary heart disease
HDL-c: High density lipoprotein cholesterol
LDL-c: Low density lipoprotein cholesterol
ELISA: Enzyme-linked immunosorbent assay
NIHSS: National Institutes of Health Stroke Scale
mRS: Modified Rankin Scale.
